# Concordance between MITS and conventional autopsies for pathological and virological diagnoses

**DOI:** 10.1007/s00414-023-03088-w

**Published:** 2023-10-14

**Authors:** Julia Schädler, Akhator Terence Azeke, Benjamin Ondruschka, Stefan Steurer, Marc Lütgehetmann, Antonia Fitzek, Dustin Möbius

**Affiliations:** 1 Institute of Legal Medicine, University Medical Center Hamburg-Eppendorf, Hamburg, Germany; 2 Department of Anatomic Pathology, Irrua Specialist Teaching Hospital, Irrua, Nigeria; 3 Institute of Pathology, University Medical Center Hamburg-Eppendorf, Hamburg, Germany; 4 Institute of Medical Microbiology, Virology, and Hygiene, University Medical Center, Hamburg-Eppendorf, Hamburg, Germany

**Keywords:** Minimally invasive tissue sampling, Conventional autopsy, COVID-19, Post-mortem ultrasound, RT-qPCR, SARS-CoV-2 specific E-gene

## Abstract

In pandemics or to further study highly contagious infectious diseases, new strategies are needed for the collection of post-mortem tissue samples to identify the pathogen as well as its morphological impact. In this study, an ultrasound-guided minimally invasive tissue sampling (MITS) protocol was developed and validated for post-mortem use. The histological and microbiological qualities of post-mortem specimens were evaluated and compared between MITS and conventional autopsy (CA) in a series of COVID-19 deaths. Thirty-six ultrasound-guided MITS were performed. In five cases more, specimens for histological and virological examination were also obtained and compared during the subsequently performed CA. Summary statistics and qualitative interpretations (positive, negative) were calculated for each organ tissue sample from MITS and CA, and target genes were determined for both human cell count (beta-globin) and virus (SARS-CoV-2 specific E gene). There are no significant differences between MITS and CA with respect to the detectability of viral load in individual organs, which is why MITS can be of utmost importance and an useful alternative, especially during outbreaks of infectious diseases.

## Introduction

Conventional autopsy (CA) is a valuable tool, especially for quality measures in healthcare [1–3], and remains gold standard in post-mortem diagnosis. However, autopsy rates have declined rapidly for a variety of reasons, such as lack of interest from physicians and relatives due to overreliance on pre-mortem diagnoses, unwillingness of relatives to consent to autopsy due to the invasiveness of the procedure, unwillingness of pathologists to perform autopsies due to shifted interests, budgetary problems, and ideological as well as ethical opposition to post-mortem examinations [4–11]. Therefore, alternative non-invasive or minimally invasive autopsy methods are being developed [12].

However, the worldwide distribution of facilities capable of performing conventional autopsies is very uneven. New strategies for the collection of post-mortem tissue samples are needed, especially in areas where emerging infections occur and where identification of the causative agent, as well as its effects on target organs, is a public health priority [13, 14] such as in pandemics.

Minimally invasive autopsy (MIA) or minimally invasive tissue sampling (MITS) has been proposed as an alternative to CA, designed as small puncture-directed diagnostic biopsies of key organs with or without imaging support. The whole body can be visualized with post-mortem ultrasound, computed tomography (CT), and magnetic resonance imaging (MRI) [15–17], and an image-guided biopsy can be performed to obtain tissue for histological and other examination [18]. In addition, CT angiography can be performed also after death [19–27]. The use of CT and CT angiography in conjunction with needle biopsy has been suggested as feasible for the diagnosis of common causes of death in the past [12, 25]. However, these techniques can only be performed in centers of excellence and require significant budgets and knowledge. Ultrasound-guided tissue aspiration has also been tested with promising results [26–32]. In the clinical setting, non-invasive or minimally invasive autopsies of fetuses, newborns, and infants [33] have gained acceptance among parents and physicians (especially compared to acceptance rates of CA) as well as political and public interest [34]. However, they are still rarely used in adult patients. The MITS methodology represents a portable, rapid, and cost-effective post-mortem technique that may be particularly useful in countries where mortality data are not readily available [27–32, 35].

The aim of the study is to develop and validate a MITS protocol established in our institution, to assess the histological and microbiological performance of the post-mortem samples, and to compare the performance of MITS and CA in a series of COVID-19 deaths.

## Materials and methods

### Study design, organizational structure, and study cohort

Starting from a game-changing first autopsy [36], numerous SARS-CoV-2 associated death case evaluations (defined as pre- and/or post-mortem confirmed SARS-CoV-2 infection of a person at any point in time) of Hamburg citizens followed at the Institute of Legal Medicine (ILM) Hamburg in collaboration with the local health authority as recently published [37]. To scrutinize the reports and in order to not overlook unknown cases, all deceased admitted to the ILM were screened for viral SARS-CoV-2 RNA using a throat swab followed by immediate RT-qPCR at the Institute of Microbiology, Virology and Hygiene, UKE, as a standard procedure [38].

Between March 2020 and December 2020, a total of 735 SARS-CoV-2 associated deaths were analyzed aiming to register (sono-) morphological data of SARS-CoV-2 associated changes, to collect tissue samples for further histological and virological evaluation and thus determine the underlying cause of death of these individuals. CA were performed in 38.5% (*n* = 283). Other death cases (5.6%; *n* = 41) were investigated by MITS. In total, 55.9% (*n* = 411) received a PMCT and 35.9% (*n* = 264) were classified by medical record review only [37]. In one subgroup among the deceased investigated, a CA followed the MITS (*n* = 5). Here, results of the CA, being still the acknowledged gold standard, were reported to the health authority. However, concordance between both methods in terms of final diagnoses and virological results was not evaluated yet scientifically.

Institutional review board approval from the independent ethics committee of the Hamburg Chamber of Physicians was obtained (reference numbers 2020–10353-BO-ff and PV7311).

Demographic (age, sex) as well as anthropometric data (height, body weight, BMI) of the deceased were collected. Moreover, typical post-mortem characteristics (post-mortem interval [PMI], post-mortem lividity, signs of decomposition) were registered at the start of post-mortem investigation.

### Examination procedure

All examinations were carried out with informed consent by relatives. According to the applied standard in the ILM during the pandemic, most of the deceased received a PMCT [39] before further investigation (Philips Brilliance 16-slice multidetector scanner, Hamburg, Germany; full-body scan: slice thickness, 1 mm; pitch, 1.5; 120 kV; 230 to 250 mAs; in addition thorax scan with higher resolution). The bodies were removed from the cooling chamber 6 to 8 h prior to ultrasonic examinations in order to warm up the soft tissue. Before the procedure, the body areal to be biopsied was cleaned using twice an antiseptic agent (both times the same agent; Octenisept®; Schülke and Mayr, Norderstedt, Germany; order no.: 32834.00.00) to minimize contamination of the collected tissue samples. During the procedure, medical staff wore single-use gowns, protective hoods, and FFP2/3 masks for individual protection. Examinations were carried out in supine position of the bodies.

**Fig. 1:**
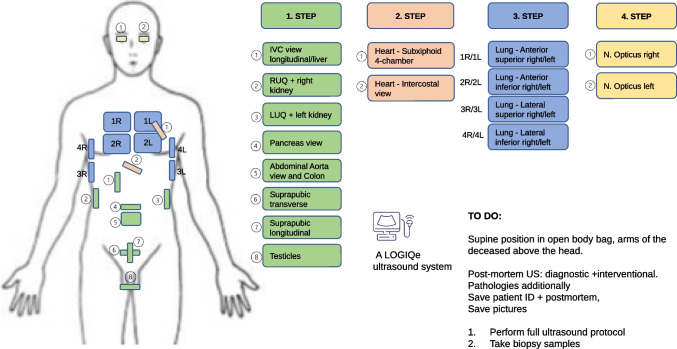
Short minimally invasive tissue sampling (MITS) protocol for COVID-19 patients (except brain) at the Institute of Legal Medicine in Hamburg, Germany. The different steps are explained. Please note the recommended position of the arms in contrast to the drawing shown here (arms of the deceased above the head). Abbreviations: IVC, inferior vena cava; RUQ, right upper quarter; LUQ, left upper quarter; R, right; L, left; ID, identification; PMI, post-mortem interval

A LOGIQe ultrasound system (LOGIQe 5417728–100, GE Medical Systems Ultrasound and Primary, Chicago, USA) was used for ultrasonic examinations of the deceased according to a standardized protocol developed in-house. We utilize our ultrasound protocol to screen for pathologies and measure all organs, including an additional assessment of the optic nerve, in order to indirectly infer the presence of cerebral edema (see Fig. 1). Next, ultrasound-guided needle punctures of the pre-defined organs (lungs, heart, liver, kidneys, spleen, prostate, or uterus) using randomly chosen semi-sterile 14G needles (SOMATEX, Berlin, Germany) were carried out. For each position, at least two cylinders for histological as well as virological processing were collected in parallel. Samples were then fixed in buffered 4% formaldehyde or stored by cryopreservation at a PMI < 72 h for virological processing. After disinfection a sealed, sterile needle was used for each position to gather samples for virological investigations first, followed by punctures for histology. In the other cases, one needle was used to cover more than one localization.

Additionally, success rates for the punctured organs were calculated. A cylinder was considered valid, when the sample consisted of at least 50% of the intended organ, microscopically, see Table 1. An acceptable rate of sampling was internally defined as 90% of all attempts.

**Table 1:** Quality control of the histology samples

Tissue	Total (*n*)	Success rates (%)	Unsuccessful sampling (%)	Mixed samples (%)	Other tissue hit	Number of biopsies (average)
Lung UL right	35	34 (97.1)	1 (2.9)	6 (17.7)	Liver, skeletal muscle, heart	2.3
Lung LL right	35	32 (91.4)	3 (8.6)	4 (12.5)	Liver, lymph node	2.5
Lung UL left	35	32 (91.4)	3 (8.6)	4 (12.5)	Heart, spleen, skeletal muscle	2.3
Lung LL left	35	29 (82.9)	6 (17.1)	1 (3.4)	Heart, liver, spleen, skeletal muscle, soft tissue	2.3
Heart	31	29 (93.5)	2 (6.5)	0 (0.0)	Liver, lung, spleen, skeletal muscle	3.1
Liver	34	34 (100.0)	0 (0.0)	0 (0.0)	No other tissue	2.8
Spleen	34	31 (91.2)	3 (8.8)	0 (0.0)	Skeletal muscle, soft tissue	2.8
Kidney right	35	34 (97.1)	1 (2.9)	0 (0.0)	Soft tissue	2.5
Kidney left	35	34 (97.1)	1 (2.9)	2 (5.9)	Skeletal muscle	2.3
Prostate/uterus	20	14 (70.0)	6 (30.0)	0 (0.0)	Soft tissue, lymph node	2.1

Table 1 shows the tissues punctured, the average number of biopsies of each organ punctured, and the associated success rates. Several biopsies were taken per organ, so that in some cases not only the desired tissue was sampled but also tissue of adjacent organs. These samples were counted as valid as long as 50% of the tissues stained where 'true'

Abbreviations: *UL* upper lobe, *LL* lower lobe

Histological criteria of COVID-19 included diffuse alveolar damage (DAD), especially hyaline membranes, and activated pneumocytes, squamous metaplasia, or organizing pneumonia [40]. Non-COVID-19 deaths were determined as acute and SARS-CoV-2 independently fatal conditions, such as myocardial infarction, ruptured arterial aneurysm, or subdural hemorrhage.

In five cases, samples for histological and virological investigations were collected also during the subsequent CA. If the detailed location was visible, tissue samples of around 0.5 × 0.5 × 2.0 cm were taken around the primary puncture localization using fresh sterile scalpels. Gathered tissues were fixed in 4% buffered formaldehyde and stored by cryopreservation as well. The forensic pathologist performed the ultrasound investigation for each organ and made diagnoses for every single case. The histological slides from the biopsies and from CA were investigated by the same pathologists independently and blindly between both forms of tissue sampling.

### Virological analysis

Post-mortem tissue was analyzed as previously described [41]. Briefly, post-mortem tissues (MITS or CA) were grinded using the Precellys 24 system (Bertin, Rockville US) and 2 ml tubes prefilled with ceramic beads (Precellys Lysing Kit) and 1 ml RNA and DNA free PCR grade water. DNA/RNA extraction was performed using the MagnaPure96 system (Roche, Mannheim, Germany) according to the manufacturer’s recommendation (input 200 µl) with whole process RNA spike in control (Roche control Kit) and a final elution volume of 100 μl. For SARS-CoV2 RNA virus quantification, the E gene assay previously published by Corman et al. [42] was used with some modification to reduce primer-primer dimer formation in tissue [41]. The one step RNA control kit was used for master and IC detection with 5 µl eluate as input as recommended by the manufacturer. β-globin PCR was performed with commercial TaqMan primer set (Thermo-Fischer, 401,846) and Roche DNA control kit; all qPCRs were run on the LightCycler480 system. *C*_*t*_ values were determined using second derivative maximum method. Quantification was performed using the appropriate standards as previously described [41]. Summary statistics and qualitative interpretation (positive, negative) were calculated for each organ tissue sample of MITS and CA, and target genes for both human cell number (beta globin; Table 2) and virus (SARS-CoV-2 specific E-gene; Table 3) were determined to confirm the quality of the tissue sample and accurately identifying the presence of a specific viral (SARS-CoV-2) infection. These molecular techniques are crucial components of the overall diagnostic process, enabling the reliable detection and characterization of viral pathogens, especially in cases where MITS techniques are employed.

**Table 2:** Summary statistics of human RNA targets for each organ

MITS – beta globin				CA – beta globin			
	Mean (copies/ml)	SD (copies/ml)	*N* total	Mean (copies/ml)	SD (copies/ml)	*N*	*p*-value
Upper lobe right	694,600.00	42,645.20	4	2,870,000.00	1,455,727.08	4	0.024*
Lower lobe right	221,066.67	89,504.71	3	2,728,000.00	562,546.59	4	< 0.001*
Upper lobe left	814,400.00	913,488.61	4	2,612,000.00	1,154,691.30	4	0.050*
Lower lobe left	658,600.00	498,708.04	4	3,922,000.00	2,472,991.17	4	0.041*
Heart	597,800.00	501,488.50	4	1,263,200.00	1,065,217.55	4	0.301
Liver	822.133.33	451,958.46	3	2,436,000.00	961,254.74	4	0.045*
Kidney right	740,000.00	657,701.53	4	2,644,000.00	989,017.02	4	0.018*
Kidney left	1,004,400.00	744,165.00	4	2,868,000.00	1,782,624.28	4	0.101
Prostate	590,400.00	736,436.39	3	3,032,000.00	824,634.06	4	0.009*
Spleen	2,073,600.00	2,125,400.32	4	4,658,000.00	2,386,954.55	4	0.157

Table 2 shows the results of summary statistics of the human RNA specific target beta globin for the biopsies and conventional autopsy tissue samples for each organ. Three biopsies did not have sufficient tissue for analysis

Abbreviations: *MITS* minimally invasive tissue sampling, *CA* conventional autopsy, *SD* standard deviation, *N* number

Statistically significant at *p* < 0.05

**Table 3:** Summary statistics of SARS-CoV-2 specific targets for each organ

MITS – Sabeco E					CA – Sabeco E				
	Mean (copies/ml)	SD (copies/ml)	Number (*N* = 36)	E-gene positive (*n* = 12)	Mean (copies/ml)	SD (copies/ml)	Number (*N* = 40)	E-gene positive (*n* = 29)	*p*-value
Upper lobe right	167,072.81	333,075.23	4	2 (50.0%)	27,383,913.01	53,715,885.07	4	4 (100.0%)	0.350
Lower lobe right	2,820,540.62	2,444,910.56	3	3 (100.0%)	6,166,054.39	12,082,171.23	4	4 (100.0%)	0.664
Upper lobe left	1,057,805.47	1,056,677.02	4	4 (100.0%)	15,842,928.16	30,930,889.09	4	4 (100.0%)	0.376
Lower lobe left	1,776,890.94	3,021,575.09	4	3 (75.0%)	1,480,057.89	1,706,392.97	4	4 (100.0%)	0.870
Heart	51,313.59	102,627.17	4	1 (25.0%)	23,823.45	34,744.00	4	2 (50.0%)	0.630
Liver	0	0	3	0 (0.0%)	2,629.60	5,226.77	4	2 (50.0%)	0.434
Kidney right	0	0	4	0 (0.0%)	11,358.86	15,677.14	4	2 (50.0%)	0.197
Kidney left	0	0	4	0 (0.0%)	10,153.32	14,621.26	4	3 (75.0%)	0.214
Prostate	0	0	3	0 (0.0%)	98,259.28	194,673.22	4	3 (75.0%)	0.432
Spleen	259.98	298.13	3	2 (66.7%)	7.02	14.04	4	1 (25.0%)	0.139

Table 3 shows the results of summary statistics of the SARS-CoV-2-specific target (Sabeco E-gene) for the biopsies and conventional autopsy tissue samples for each organ. Three biopsies did not have sufficient tissue for analysis. No statistically significant differences were present

*Abbreviations*: *MITS* minimally invasive tissue sampling, *CA* conventional autopsy, *SD* standard deviation, *N* number

### Statistical analysis

Statistical analysis was performed descriptively using Microsoft Excel (version 16.16, Microsoft Corporation, Redmond, USA) and GraphPad Prism® (version 8.0, GraphPad Software Inc., La Jolla, USA). Variables were described as percentages, means, and standard deviations (SD). The assumption of a normal distribution was checked by Shapiro–Wilk-test and compared by the *t* test. Graphical representation of results was performed using the GraphPad Prism® statistical software (version 8.0, GraphPad Software Inc., La Jolla, USA) and Microsoft Power Point 2016.

## Results

### Demographic characteristics

We performed 36 ultrasound-guided MITS. 91.7% (*n* = 33) of the examined bodies had a positive post-mortem RT-qPCR nasopharyngeal swab for SARS-CoV-2. The mean PMI between death and investigation was 3.3 (SD ± 2.3) days. Men comprised 66.7% (*n* = 24) of all biopsy patients. Mean age was 80.2 (SD ± 13.0) years, and mean BMI was 25.6 (SD ± 6.1) kg/m^2^.

### MITS results in high success rates of organ samples

On average, 2.1 to 3.1 biopsies could be taken per organ for further histological investigation. Tissue sampling was not attempted for all organs in the cases reviewed because in some fatalities, organs were missing or access to the appropriate regions was not available for ultrasound guiding (see Table 1). Histologically confirmed, the liver was aimed in all cases. The left lower lobe of the lung was only on target in 82.9% (*n* = 29/35). Only the prostate and uterus (70.0%; *n* = 14/20) were more difficult to puncture successfully, overall. The accuracy of all other punctured organs was above 90.0% (see Table 1). In the biopsies from the lungs and left kidney, there was a small number of microscope slides showing tissue of the originally intended organ as well as tissue of adjacent organs, skeletal muscle, or soft tissue (Table 1) like a representation of the needle tract. These samples were still counted as regular biopsies, if more than 50% of the intended organ was visible.

In 20 (58.8%) cases of the right and in 19 (55.9%) cases of the left kidney, more than 10 glomeruli were counted by light microscopy (see Table 4). Histologically, in all biopsies of the liver except two, portal fields and central veins were always observed to illustrate typical histoarchitecture of this organ.

**Table 4:** Numbers of glomeruli in renal biopsies

Glomeruli	Right kidney *n* (%)	Left kidney *n* (%)
< 5	7 (20.6)	7 (20.6)
5–10	7 (20.6)	8 (23.5)
> 10	20 (58.8)	19 (55.9)

Table 4 shows the number of counted glomeruli in all biopsies of the right and left kidney. In total, 34 renal biopsies were performed for each side

### MITS lung samples are diagnostically useful, while others have failed to reveal the main underlying pathology

We performed five MITS gathering needle biopsies of ten different locations followed by CA in post-mortem RT-qPCR-positive decedents. During CA, tissue samples were collected near the previously ultrasound-guided punctured regions of the organs in question. All five decedents were men. Mean age and BMI were 70.0 (SD ± 16.9) years and 30.2 (SD ± 9.0) kg/m^2^, respectively. The mean PMI was 2.4 (SD ± 0.5) days. In only two of all possible locations in the five cases (2/50; 4.0%), the aimed tissue was not successfully acquired. This corresponds to a success rate of 96.0% for the examined MITS cases. However, in one of these cases, there was a second biopsy which shows the correct tissue (adjusted accuracy 98.0%). Comparison of the main histologic findings showed missing pathological aspects in either the biopsies or the conventionally taken tissues in six different organs within all 50 samples or locations. For example, in case 4, CA histology diagnosed fibrinous pericarditis. In contrast, the cardiac muscle tissue of the biopsy appeared normal, but pericardium was not visualized nor stained in this slide. Compared to CA, a concordance rate in main diagnosis of up to 87.5% was reached. Histopathological evaluation of the MITS lung samples showed typical pathologies which were similar to those described in published autopsy studies [43–47]. For more detail, see Table 5. The concordance between presumed cause of death after histological examination including findings of post-mortem ultrasound and CA findings was up to 80% (see Table 5).

**Table 5:** Comparison of the main histologic findings and presumed cause of death after histological examination of biopsies and tissue samples of conventional autopsies

Tissue	Case 1		Case 2		Case 3		Case 4		Case 5	
	MITS	CA	MITS	CA	MITS	CA	MITS	CA	MITS	CA
Lung UL right	Normal	Normal	DAD	DAD	Hyperemia	Acute edema	DAD	DAD	Emphysema	Emphysema, **fibrosis**
Lung LL right	Organizing pneumonia	Organizing pneumonia	DAD	DAD	Acute edema, **liver**	Acute edema	DAD	DAD	**Normal**	**Emphysema**
Lung UL left	Acute edema	Acute edema	Acute lung injury	Acute lung injury	Acute edema	Acute edema	DAD	DAD	**Acute edema**	**Fibrosis**
Lung LL left	Pneumonia	Pneumonia	DAD	DAD	Acute edema	Acute edema	DAD	DAD	Acute edema	**Emphysema,** acute edema
Heart	**Liver**	**Normal**	Fibrosis	Fibrosis	Myocardial infarction, fibrosis	Myocardial infarction, fibrosis	Normal*	**Fibrinous pericarditis**	Fibrosis, necrosis	Fibrosis, necrosis
Liver	Mild steatosis	Mild steatosis	Moderate steatosis	Moderate steatosis	Mild steatosis	Mild steatosis	Moderate steatosis	Moderate steatosis	Normal	Normal
Spleen	Chronic congestion	Chronic congestion	Chronic congestion	Chronic congestion	Chronic congestion	Chronic congestion	**Normal**	**Chronic congestion**	Chronic congestion	Chronic congestion
Kidney right	Normal	Normal	Normal	Normal	Normal	Normal	Normal	Normal	Arterio-arteriolosclerosis	Arterio-arteriolosclerosis
Kidney left	Normal	Normal	Normal	Normal	Normal	Normal	Normal	Normal	Normal	Normal
Prostate	BPH	BPH	BPH	BPH	BPH	BPH	BPH	BPH	BPH	BPH
Presumed cause of death	Organizing pneumonia, subacute lung injury	Sepsis, urocystitis, organizing pneumonia	DAD, acute lung injury	Pneumonia, DAD	Myocardial infarction	Ruptured myocardial infarction	Pneumonia, DAD	Pneumonia, DAD	Presumed cardial decompensation	Cardial decompensation

Table 5 compares the main histologic findings (main diagnosis) in biopsies and tissue samples of conventional autopsies. Furthermore, it shows differences in the presumed cause of death after histological examination including findings of post-mortem ultrasound and conventional autopsy findings. All tissue samples were formalin-fixed and stained with hematoxylin eosin. Differences are highlighted in bold. In two biopsies, the aimed tissue was not successfully sampled or were mixed samples (Cases 1 and 3), and in six organ samples, another pathology aspect was missing in the histology of the biopsy

*Abbreviations*: *UL* upper lobe, *LL* lower lobe, *MITS* minimally invasive tissue sampling, *CA* conventional autopsy, *DAD* diffuse alveolar damage, *BPH* benign prostate hyperplasia

*Pericardial tissue was not biopsied; only myocardial layers were examined

### Virological assessment can be applied in MITS without relevant loss of diagnostic power

**Fig. 2:**
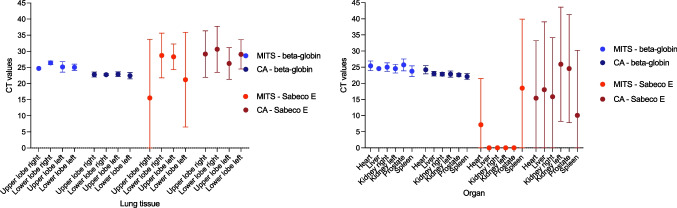
Human RNA (beta globin) and SARS-CoV-2-specific (Sabeco E) *C*_*t*_ values obtained for each biopsy sample and autopsy tissue sample. Abbreviations: MITS, minimally invasive tissue sampling; CA, conventional autopsy. Sabeco E = virus specific E-gene. *C*_*t*_ = cycle threshold

**Fig. 3:**
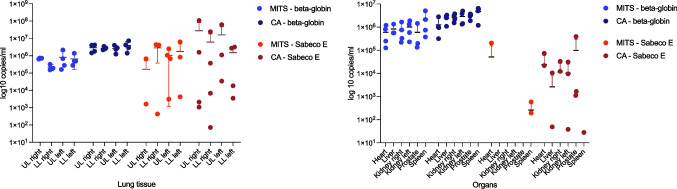
Human RNA (beta globin) and SARS-CoV-2-specific (Sabeco E) copies per milliliter (copies/ml) obtained for each biopsy sample and autopsy tissue sample, if detectable. The results are presented in logarithmic form of copies/ml. Abbreviations: UL, upper lobe; LL, lower lobe; MITS, minimally invasive tissue sampling; CA, conventional autopsy

In four of the above-mentioned cases, a microbiological diagnostic was performed. Therefore, a total of 15 biopsies of the lungs and 38 biopsies of the heart, liver, both kidneys, spleen, and prostate/uterus were taken. First, *C*_*t*_-values for human beta globin in CA tissue samples were in total lower (i.e., more infectious) than in tissue samples of MITS (see Fig. 2), but there was a main difference in sample size of biopsy (14G)- and autopsy samples (autopsy samples typically approximately 1 × 1 × 0.5 cm vs. MITS samples 0.4 × 0.4 × 0.4 cm). The volume-adjusted comparison of tissue samples from both groups shows that there are no differences in quality between tissue samples from the heart, left kidney, and spleen by comparing the human target gene beta globin. For all other organs, there were statistically significant differences (Table 2). The diagnostically crucial number of 1000 copies/ml was exceeded in both types of samples. The results are represented in Fig. 3 in its logarithmic form.

Concerning virus-specific E-gene, there were no significant differences between both groups (Table 3). All four patients had high levels of virus load in their lung samples of both MITS (*n* = 15) and CA (*n* = 16). In detail, no virus-specific gene could be detected in 3/15 lung tissue biopsy samples but was detected in the further tissue samples obtained at autopsy. The virus load varied between organs (heart, liver, prostate, spleen, and kidney samples). There was no virus detected in 18/23 non-lung biopsies. In the corresponding CA specimens, virus detection was partially successful in these cases, but in low copy numbers.

In these cases, contamination of the CA specimens may have occurred. Contamination by the environment, on the other hand, was tried to be minimized in the case of needle biopsies by disinfection of the body part prior to the procedure.

In the CA tissue samples, no virus was detectable in 11/24 organ samples. In the samples from one descendent, no virus-specific genes could be detected in any biopsy; the corresponding samples obtained during CA were also negative, except in the kidney samples with corresponding *C*_*t*_ values above 30.

## Discussion

This study demonstrates the concordance of MITS and CA in the quality of tissue samples as well as in the main diagnosis and cause of death in deceased patients infected with SARS-CoV-2. Our main results indicate up to 100.0% histological confirmed success rates in MITS samples, except the success rates of the left lower lobe of the lung and the prostate/uterus. Due to bigger samples and more cross-section of small vessels, *C*_*t*_-values for human beta globin in tissue samples of CA were in total lower than in tissue samples of MITS. However, the volume-adjusted comparison of tissue samples from both groups shows that there were no differences in quality between tissue samples from the heart, left kidney, and spleen by comparing the human target gene beta globin. Also, concerning virus-specific E-gene RNA, there were no significant differences between both groups.

### Minimally invasive tissue sampling using ultrasound

Beginning in 1998, Fariña et al. published the first report on post-mortem examinations using ultrasound-guided biopsies. In the next years, further comparative studies followed. These studies mainly focused on determination of the cause of death and the associated detection of bacterial or viral infections by recording the ultrasound findings followed by a subsequent histological and microbiological evaluation. In different comparative studies between MITS results and CA diagnoses, concordance rates between 83 and 92% could be observed with regard to previous diseases and cause of death, which goes in line with the results of our study [26, 28–32, 48].

The here given rates of successful tissue sampling ranged from 70% (prostate) to 100% (liver), as already reported in other studies [26, 49, 50]. Thus, it can be concluded that MITS is a reliable method for taking tissue samples of specific organs. For the left lower lobe of the lung, valid samples could only be obtained in 82.9% of the cases not surpassing our pre-defined quality level. Possible reasons can be a reduced image quality due to air-related artifacts, proximity to the adjacent heart or different heights of the diaphragm.

Concerning the criteria for a clinically obtained kidney biopsy, there is disagreement on how many glomeruli, tubules, and vessels are required for an accurate histological diagnosis of kidney disease [51, 52]. As a directive, several authors suggest that native kidney samples should contain at least 10 to 20 glomeruli in their entirety to exclude focal disease processes and allow accurate assessment of the extent of glomerular involvement [53, 54]. We reached these reference value in approximately 80% of post-mortem renal samples, although autolytically altered and therefore non-assessable glomeruli were strictly not counted.

Ultrasound offers the possibility of guided tissue sampling on the one hand and its use as an imaging tool for morphological diagnoses on the other hand. For example, the depiction of fluids (e.g., pleural effusion, pericardial tamponade) or metastases can give hints for underlying pathologies and contribute to determine the cause of death [55].

### Main diagnosis / presumed cause of death

In clinical routine, ultrasound is commonly used and educational trainings for physicians exist. To date, in legal medicine, there is no common practice using ultrasound, though valuable tendencies can be observed [56]. Nevertheless, even when using standard protocols for investigation, sonomorphological diagnostics is influenced by experience and subjective assessment and depends on the cadaveric condition [57, 58, 58–60].

Superordinately, MITS can be used to obtain comparable results to CA with respect to the cause of death. After interpretation of the findings from histological examinations and post-mortem ultrasound examination with integration of the clinical findings, similar results were obtained in all five cases after both MITS and CA. Of course, CA is much more efficient. For example, in case 1, urocystitis next to pneumonia could not be detected by MITS. Case 3 shows a pericardial effusion in MITS; bloody or serous effusion cannot be differentiated by ultrasound (puncture of the effusion was not successful); the localization of the myocardial infarction was randomly hit with the punch biopsies, since targeted imaging of myocardial infarction and then aimed ultrasound-guided puncture is not possible with routine methods. The more deeper a tissue alteration of the lungs, the lower is the chance to diagnose it using MITS and histology. Another drawback regarding the presumed causes of death is the well-protected brain masking any cerebral or spinal disease for ultrasound in this study. Only in case 4, a significant intracerebral finding (hemorrhage during ECMO therapy) was described during autopsy. To further improve the MITS procedure also for cerebral and spinal pathologies, the authors are currently in the process of establishing an intracranial examination and sampling method through a cranial minimally invasive access route.

### Virological examinations

Human beta globin is commonly used to monitor quantity of human cells extracted from a given sample [61]. In biopsy samples, significantly less total beta globin could be detected than in the corresponding samples from the CA (Table 2; Figs. 2 and 3).

In the CA samples, a significantly lower *C*_*t*_ value, corresponding to a higher viral load, could be detected than in the corresponding MITS samples. However, it should be noted that the CA samples contain more tissue than the MITS samples processed, so that a significant difference in analysis can be seen here due to the sample quantity but not necessarily quality. On the other hand, considering the detectability of the viral load in the individual organs, there are no significant differences in overall detection between both methods MITS and CA. Detection of SARS-CoV-2 in examined tissues succeeds similar to other infectious diseases especially in lung tissue [29, 48, 62]. The viral load as well as the *C*_*t*_ values are similarly high for both investigation modes. Organ-specific viral loads, as they have already been described several times before [41, 63, 64], are also reflected in our collective. In a large amount of biopsies, no virus was detected in the kidney, liver, spleen, or prostate. In the corresponding CA specimens, virus detection was partially successful in these cases. However, the copy number was rather low here. In these cases, contamination of the CA specimens may have occurred (e.g., organs lying nearby at the autopsy table). Contamination by the environment, on the other hand, was tried to be minimized in the case of needle biopsies by disinfection of the body prior to the procedure.

## Limitations

Our study included only a relatively small number of specimens in which direct comparison of sample quality between MITS and CA tissue samples was examined virologically which was due to economic reasons. Due to the method, significantly smaller tissue samples could be taken in MITS compared to CA despite the use of larger needles. Regarding ultrasound image quality, numerous influences have already been demonstrated in previous studies and case series [55, 65]. In particular, post-mortem changes of the body, such as progressive decomposition and the associated formation of gas, a longer PMI, a cooled down body, or even an obese nutritional state, led to lower ultrasound quality or to artifacts and thus had a significant influence on the accuracy of tissue sampling and diagnosis. In case of very air-rich lungs, such as can be seen in emphysema, it was also difficult to obtain sufficient sample material by needle biopsy. Moreover, the verifiability of the correct tissue in needle biopsies for virology assessment was not given. Finally, not all solid organs and soft tissues were sampled in this study and some of them might be more difficult to hit rendering potential differences in other probes possibly.

## Conclusion

Considering the detectability of the viral load in individual organs, there are no significant differences between MITS and CA, why MITS may be of upmost importance and a useful alternative, especially in infectious disease outbreaks. As many outbreaks occur in settings, where the feasibility and general acceptance of CA remain challenging, MITS should be implemented in all clinical settings. It is important to keep in mind, however, that these alternative methods are still less accurate than the reference standard, so it is crucial to perform image-guided tissue biopsies (and therefore a minimally invasive autopsy) for accurate diagnosis. Furthermore, the utilization of MITS in future outbreaks may accelerate the generation of scientific knowledge around reasons and the cause of death of emerging infections.

## Data Availability

The datasets generated during and/or analyzed during the current study are available from the corresponding author on reasonable request.

## References

[1] Kircher T, Nelson J, Burdo H (1985). The autopsy as a measure of accuracy of the death certificate. N Engl J Med.

[2] Winters B, Custer J, Galvagno SM, Colantuoni E, Kapoor SG, Lee H (2012). Diagnostic errors in the intensive care unit: a systematic review of autopsy studies. BMJ Qual Saf.

[3] Shojania KG, Burton EC, McDonald KM, Goldman L (2003). Changes in rates of autopsy-detected diagnostic errors over time: a systematic review. JAMA.

[4] Burton JL, Underwood J (2007). Clinical, educational, and epidemiological value of autopsy. Lancet.

[5] Shojania KG, Burton EC (2008). The vanishing nonforensic autopsy. N Engl J Med.

[6] Ayoub T, Chow J (2008). The conventional autopsy in modern medicine. J R Soc Med.

[7] Oluwasola OA, Fawole OI, Otegbayo AJ, Ogun GO, Adebamowo CA, Bamigboye AE (2009). The autopsy: knowledge, attitude, and perceptions of doctors and relatives of the deceased. Arch Pathol Lab Med.

[8] Blokker BM, Weustink AC, Hunink MGM, Oosterhuis JW (2016). Autopsy of adult patients deceased in an academic hospital: considerations of doctors and next-of-kin in the consent process. PLoS One.

[9] Blokker BM, Weustink AC, Hunink MGM, Oosterhuis JW (2017). Autopsy rates in the Netherlands: 35 years of decline. PLoS One.

[10] Brown HG (1990). Perceptions of the autopsy: views from the lay public and program proposals. Hum Pathol.

[11] Council on Scientific Affairs AMA, Chicago (1987). Autopsy: a comprehensive review of current issues. JAMA.

[12] Blokker BM, Wagensveld IM, Weustink AC, Oosterhuis JW, Hunink MGM (2016). Non-invasive or minimally invasive autopsy compared to conventional autopsy of suspected natural deaths in adults: a systematic review. Eur Radiol.

[13] Perkins MD, Dye C, Balasegaram M, Bréchot C, Mombouli JV, Røttingen JA (2017). Diagnostic preparedness for infectious disease outbreaks. Lancet.

[14] Pigott DM, Deshpande A, Letourneau I, Morozoff C, Reiner RC, Kraemer MUG (2017). Local, national, and regional viral haemorrhagic fever pandemic potential in Africa: a multistage analysis. Lancet (London, England).

[15] Roberts IS, Benamore RE, Benbow EW, Lee SH, Harris JN, Jackson A (2012). Post-mortem imaging as an alternative to autopsy in the diagnosis of adult deaths: a validation study. Lancet.

[16] Westphal SE, Apitzsch J, Penzkofer T, Mahnken AH, Knüchel R (2012). Virtual CT autopsy in clinical pathology: feasibility in clinical autopsies. Virchows Arch.

[17] Wichmann D, Obbelode F, Vogel H, Hoepker WW, Nierhaus A, Braune S (2012). Virtual autopsy as an alternative to traditional medical autopsy in the intensive care unit: a prospective cohort study. Ann Intern Med.

[18] Weustink AC, Hunink MG, van Dijke CF, Renken NS, Krestin GP, Oosterhuis JW (2009). Minimally invasive autopsy: an alternative to conventional autopsy?. Radiology.

[19] Rutty GN, Morgan B, Robinson C, Raj V, Pakkal M, Amoroso J (2017). Diagnostic accuracy of post-mortem CT with targeted coronary angiography versus autopsy for coroner-requested post-mortem investigations: a prospective, masked, comparison study. Lancet.

[20] Michaud K, Grabherr S, Jackowski C, Bollmann MD, Doenz F, Mangin P (2014). Postmortem imaging of sudden cardiac death. Int J Legal Med.

[21] Westphal SE, Apitzsch JC, Penzkofer T, Kuhl CK, Mahnken AH, Knüchel R (2014). Contrast-enhanced postmortem computed tomography in clinical pathology: enhanced value of 20 clinical autopsies. Hum Pathol.

[22] Wichmann D, Heinemann A, Weinberg C, Vogel H, Hoepker WW, Grabherr S (2014). Virtual autopsy with multiphase postmortem computed tomographic angiography versus traditional medical autopsy to investigate unexpected deaths of hospitalized patients: a cohort study. Ann Intern Med.

[23] Ross SG, Thali MJ, Bolliger S, Germerott T, Ruder TD, Flach PM (2012). Sudden death after chest pain: feasibility of virtual autopsy with postmortem CT angiography and biopsy. Radiology.

[24] Grabherr S, Doenz F, Steger B, Dirnhofer R, Dominguez A, Sollberger B (2011). Multi-phase post-mortem CT angiography: development of a standardized protocol. Int J Legal Med.

[25] Bolliger SA, Filograna L, Spendlove D, Thali MJ, Dirnhofer S, Ross S (2010). Postmortem imaging-guided biopsy as an adjuvant to minimally invasive autopsy with CT and postmortem angiography: a feasibility study. AJR Am J Roentgenol.

[26] Fariña J, Millana C, Fdez-Aceñero MJ, Furió V, Aragoncillo P, Martín VG (2002). Ultrasonographic autopsy (echopsy): a new autopsy technique. Virchows Arch.

[27] Cox JA, Lukande RL, Kalungi S, Van Marck E, Van de Vijver K, Kambugu A (2014). Needle autopsy to establish the cause of death in HIV-infected hospitalized adults in Uganda: a comparison to complete autopsy. J Acquir Immune Defic Syndr.

[28] Castillo P, Ussene E, Ismail MR, Jordao D, Lovane L, Carrilho C (2015). Pathological methods applied to the investigation of causes of death in developing countries: minimally invasive autopsy approach. PLoS One.

[29] Martínez MJ, Massora S, Mandomando I, Ussene E, Jordao D, Lovane L (2016). Infectious cause of death determination using minimally invasive autopsies in developing countries. Diagn Microbiol Infect Dis.

[30] Castillo P, Martínez MJ, Ussene E, Jordao D, Lovane L, Ismail MR (2016). Validity of a minimally invasive autopsy for cause of death determination in adults in Mozambique: an observational study. PLoS Med.

[31] Castillo P, Hurtado JC, Martínez MJ, Jordao D, Lovane L, Ismail MR (2017). Validity of a minimally invasive autopsy for cause of death determination in maternal deaths in Mozambique: an observational study. PLOS Med.

[32] Bassat Q, Castillo P, Martínez MJ, Jordao D, Lovane L, Hurtado JC (2017). Validity of a minimally invasive autopsy tool for cause of death determination in pediatric deaths in Mozambique: an observational study. PLoS Med.

[33] Karat AS, Tlali M, Fielding KL, Charalambous S, Chihota VN, Churchyard GJ (2017). Measuring mortality due to HIV-associated tuberculosis among adults in South Africa: comparing verbal autopsy, minimally-invasive autopsy, and research data. PLoS One.

[34] Martines RB, Bhatnagar J, de Oliveira Ramos AM, Davi HP, Iglezias SD, Kanamura CT (2016). Pathology of congenital Zika syndrome in Brazil: a case series. Lancet.

[35] Bassat Q, Ordi J, Vila J, Ismail MR, Carrilho C, Lacerda M (2013). Development of a post-mortem procedure to reduce the uncertainty regarding causes of death in developing countries. Lancet Glob Health.

[36] Fitzek A, Sperhake J, Edler C, Schröder AS, Heinemann A, Heinrich F (2020). Evidence for systematic autopsies in COVID-19 positive deceased: case report of the first German investigated COVID-19 death. Rechtsmedizin (Berl).

[37] Fitzek A, Schädler J, Dietz E, Ron A, Gerling M, Kammal AL (2021). Prospective postmortem evaluation of 735 consecutive SARS-CoV-2-associated death cases. Sci Rep.

[38] Pfefferle S, Reucher S, Nörz D, Lütgehetmann M (2020). Evaluation of a quantitative RT-PCR assay for the detection of the emerging coronavirus SARS-CoV-2 using a high throughput system. Euro Surveill.

[39] Kniep I, Lutter M, Ron A, Edler C, Püschel K, Ittrich H (2020). Postmortale Bildgebung der Lunge bei COVID-19-Todesfällen. Radiologe.

[40] Dettmeyer R, Lasczkowski G, Weber A, Wolter T, Kernbach-Wighton G (2020). Histopathological findings following SARS-CoV-2 infection with and without treatment - report of three autopsies. Rechtsmedizin (Berl).

[41] Puelles VG, Lutgehetmann M, Lindenmeyer MT, Sperhake JP, Wong MN, Allweiss L (2020). Multiorgan and renal tropism of SARS-CoV-2. N Engl J Med.

[42] Corman VM, Landt O, Kaiser M, Molenkamp R, Meijer A, Chu DK et al (2020) Detection of 2019 novel coronavirus (2019-nCoV) by real-time RT-PCR. Euro Surveill 25:2000045. 10.2807/1560-7917.Es.2020.25.3.2000045 10.2807/1560-7917.ES.2020.25.3.2000045 PMC6988269 31992387

[43] Edler C, Schröder AS, Aepfelbacher M, Fitzek A, Heinemann A, Heinrich F (2020). Dying with SARS-CoV-2 infection-an autopsy study of the first consecutive 80 cases in Hamburg, Germany. Int J Legal Med.

[44] Wichmann D, Sperhake JP, Lutgehetmann M, Steurer S, Edler C, Heinemann A (2020). Autopsy findings and venous thromboembolism in patients with COVID-19: a prospective cohort study. Ann Intern Med.

[45] Kommoss FKF, Schwab C, Tavernar L, Schreck J, Wagner WL, Merle U (2020). The pathology of severe COVID-19-related lung damage. Dtsch Arztebl Int.

[46] Konopka KE, Nguyen T, Jentzen JM, Rayes O, Schmidt CJ, Wilson AM (2020). Diffuse alveolar damage (DAD) resulting from coronavirus disease 2019 infection is morphologically indistinguishable from other causes of DAD. Histopathology.

[47] Ackermann M, Verleden SE, Kuehnel M, Haverich A, Welte T, Laenger F (2020). Pulmonary vascular endothelialitis, thrombosis, and angiogenesis in Covid-19. N Engl J Med.

[48] Duarte-Neto AN, Monteiro RAA, Johnsson J, Cunha MDP, Pour SZ, Saraiva AC (2019). Ultrasound-guided minimally invasive autopsy as a tool for rapid post-mortem diagnosis in the 2018 Sao Paulo yellow fever epidemic: correlation with conventional autopsy. PLoS Negl Trop Dis.

[49] Brook OR, Piper KG, Mercado NB, Gebre MS, Barouch DH, Busman-Sahay K (2021). Feasibility and safety of ultrasound-guided minimally invasive autopsy in COVID-19 patients. Abdom Radiol (NY).

[50] Li Y, Wu J, Wang S, Li X, Zhou J, Huang B (2021). Progression to fibrosing diffuse alveolar damage in a series of 30 minimally invasive autopsies with COVID-19 pneumonia in Wuhan, China. Histopathology.

[51] Chung S, Koh ES, Kim SJ, Yoon HE, Park CW, Chang YS (2014). Safety and tissue yield for percutaneous native kidney biopsy according to practitioner and ultrasound technique. BMC Nephrol.

[52] Kim D, Kim H, Shin G, Ku S, Ma K, Shin S (1998). A randomized, prospective, comparative study of manual and automated renal biopsies. Am J Kidney Dis.

[53] Constantin A, Brisson ML, Kwan J, Proulx F (2010). Percutaneous US-guided renal biopsy: a retrospective study comparing the 16-gauge end-cut and 14-gauge side-notch needles. J Vasc Interv Radiol.

[54] Xie W, Xu J, Xie Y, Lin Z, Xu X, Zhang X (2020). Adequacy and complication rates of percutaneous renal biopsy with 18- vs. 16-gauge needles in native kidneys in Chinese individuals. BMC Nephrol.

[55] Möbius D, Fitzek A, Hammer N, Heinemann A, Ron A, Schädler J (2021). Ultrasound in legal medicine-a missed opportunity or simply too late? A narrative review of ultrasonic applications in forensic contexts. Int J Legal Med.

[56] Püschel K, Heinemann A, Dietz E, Hellwinkel O, Henners D, Fitzek A (2020). New developments and possibilities in the field of post-mortem medicine mortui vivos docent. Rechtsmedizin.

[57] Valentin L (2006). Minimum training recommendations for the practice of medical ultrasound. Education, practical standards committee, european federation of societies for ultrasound in medicine and biology. Ultraschall Med.

[58] Bhagra A, Tierney DM, Sekiguchi H, Soni NJ (2016). Point-of-care ultrasonography for primary care physicians and general internists. Mayo Clin Proc.

[59] Nicholls D, Sweet L, Hyett J (2014). Psychomotor skills in medical ultrasound imaging: an analysis of the core skill set. J Ultrasound Med.

[60] Ostergaard ML, Rue Nielsen K, Albrecht-Beste E, Kjaer Ersboll A, Konge L, Bachmann Nielsen M (2019). Simulator training improves ultrasound scanning performance on patients: a randomized controlled trial. Eur Radiol.

[61] Buchta C, Camp JV, Jovanovic J, Chiba P, Puchhammer-Stockl E, Mayerhofer M (2021). The versatility of external quality assessment for the surveillance of laboratory and in vitro diagnostic performance: SARS-CoV-2 viral genome detection in Austria. Clin Chem Lab Med.

[62] Saegeman V, Cohen MC, Burton JL, Martinez MJ, Rakislova N, Offiah AC (2021). Microbiology in minimally invasive autopsy: best techniques to detect infection. ESGFOR (ESCMID study group of forensic and post-mortem microbiology) guidelines. Forensic Sci Med Pathol.

[63] Remmelink M, De Mendonca R, D’Haene N, De Clercq S, Verocq C, Lebrun L (2020). Unspecific post-mortem findings despite multiorgan viral spread in COVID-19 patients. Crit Care.

[64] Bradley BT, Maioli H, Johnston R, Chaudhry I, Fink SL, Xu H (2020). Histopathology and ultrastructural findings of fatal COVID-19 infections in Washington State: a case series. Lancet.

[65] Uchigasaki S, Tsokos M (2006). Postmortem ultrasound imaging in forensic pathology. Forensic pathology reviews.

